# Crops Responses to Mite Infestation: It's Time to Look at Plant Tolerance to Meet the Farmers' Needs

**DOI:** 10.3389/fpls.2018.00556

**Published:** 2018-04-24

**Authors:** Raul A. Sperotto, Giseli Buffon, Joséli Schwambach, Felipe K. Ricachenevsky

**Affiliations:** ^1^Graduate Program in Biotechnology, University of Taquari Valley, Lajeado, Brazil; ^2^Biological Sciences and Health Center, University of Taquari Valley, Lajeado, Brazil; ^3^Graduate Program in Biotechnology, University of Caxias do Sul, Caxias do Sul, Brazil; ^4^Graduate Program in Agrobiology, Federal University of Santa Maria, Santa Maria, Brazil; ^5^Graduate Program in Cell and Molecular Biology, Federal University of Rio Grande do Sul, Porto Alegre, Brazil

**Keywords:** antibiosis, antixenosis, plant defense, tolerance, herbivore performance

It is common to see crop farmers struggling to solve herbivore infestations. Normally, worry starts when herbivore population increases and leaf damage is apparent. Whatever the treatment chosen by the farmer (chemical or biological) to control it, yield and productivity are the final and most important characteristics they would like to see unaffected by herbivory. Certainly, farmers would be less interested in percentage of leaf damage or mite population increase, which are useful but limited approximations of tolerance, than maintenance of crop production even under infested conditions. If a crop can stand infestation and/or larger mite populations while still producing the same seed set, the crop is, from a farmer standpoint, tolerant. On the other hand, academic studies on the field of plant-herbivore interaction are still based on resistance mechanisms in vegetative tissues and herbivore performance, rather than the plant reproductive success. We should be aware that genetic breeding for tolerance should focus on comparing seed production when dealing with most crops, not indirect measures such as leaf damage or mite population dynamics.

## Resistance/tolerance mechanisms

The interactions between herbivores and plant hosts result from an elaborate evolutionary interplay: plants have developed strategies to arrest attackers and reduce pest fitness as a defense against herbivory, while herbivores have evolved mechanisms to overcome that (Rioja et al., 2017). Such defense strategies include several modifications that reduce the negative impact of herbivores on a plant's reproductive success (i.e., the production of fertile offspring) and increase the plant's fitness (i.e., its contribution to the gene pool of the next generation) as a function of herbivory (Erb, 2018). Plants that efficiently and effectively use these defense strategies are called “pest-resistant” (which can be broadly classified into two different mechanisms, antibiosis and antixenosis - Stenberg and Muola, 2017), or “pest-tolerant” (Mitchell et al., 2016). Antibiosis mechanisms affect pest biology in a deleterious manner (Peterson et al., 2017), decreasing herbivore fitness or performance (e.g., fertility rate or larval development time - Stenberg and Muola, 2017). Antixenosis mechanisms direct a pest away from the plant (Peterson et al., 2017), decreasing the herbivore presence (number of eggs, larvae, or adults) and, consequently, the herbivore damage (e.g., percentage of leaf area removed - Stenberg and Muola, 2017). On the contrary, tolerance mechanisms allow the plants to withstand pest injury and produce acceptable yields, maintaining the fitness under stressful conditions, without affecting pest biology or behavior, which creates little selective pressure on pest populations and therefore does not generate resistant variants to the tolerant plants (Peterson et al., 2017).

## Tolerance: much less studied than antibiosis or antixenosis

Recently, a tricky and artful question was made by Peterson et al. (2017): is tolerance the forgotten child of plant resistance? This questioning came from the fact that it has received the least attention of the three types of plant defenses (or the three types of plant resistance, according to some authors that consider tolerance as the third type of plant resistance). According to Erb (2018), most plant defenses are still characterized by proximate variables such as herbivore performance or plant damage rather than actual fitness, which means that antibiosis and antixenosis (resistance subtypes) are more commonly used than tolerance to describe plant behavior against herbivorous pests. This is evidenced by the data presented in Table 1. Since 2009, we found 25 articles describing phytophagous mite interaction with crop species, and only four analyzed plant yield under infested condition (Karmakar, 2009; Vichitbandha and Chandrapatya, 2011; Nyoike and Liburd, 2013; Warabieda, 2015). The most common group of measures to assess defenses are herbivore performance traits, including mite population, survival, development and oviposition rates, along with leaf damage (Table 1). Peterson et al., 2017) list five reasons why tolerance has not been developed as successfully as antibiosis and antixenosis: (1) tolerance is difficult to identify, and the mechanisms conferring it are poorly understood; (2) the genetics of tolerance is mostly unknown; (3) high-throughput phenotyping methods for large-scale screening of tolerance are still missing; (4) most of the entomologists are interested in mechanisms which affect pest biology, not plant biology (highlighting the need for interdisciplinary research between plant scientists and entomologists); and (5) plant resistance efforts are still directed at controlling pest populations rather than managing plant stress.

**Table 1:** Studies on crop-mite interactions since 2009.

**Crop**	**Mite species**	**Analyzed trait**	**Yield analysis**	**References**
Rice	*Steneotarsonemus spinki*	Mite population, leaf sheath and grain damage, and yield loss	X	Karmakar, 2009
Maize	*Tetranychus urticae* and *Brevipalpus chilensis*	Mite population	–	Carrillo et al., 2011
Tomato	*Tetranychus evansi*	Mite survival, development and oviposition rates	–	Onyambus et al., 2011
Chili	*Polyphagotarsonemus latus*	Shoot damage and yield loss	X	Vichitbandha and Chandrapatya, 2011
Tomato	*Tetranychus urticae* and *Tetranychus evansi*	Mite survival, development and oviposition rates	–	Bleeker et al., 2012
Strawberry	*Tetranychus urticae*	Mite population and yield loss	X	Nyoike and Liburd, 2013
Tomato	*Tetranychus urticae*	Leaf damage and mite population	–	Salinas et al., 2013
Maize and tomato	*Tetranychus urticae*	Mite population	–	Szczepaniec et al., 2013
Grapevine	*Colomerus vitis*	Leaf damage and mite population	–	Khederi et al., 2014
Wheat	*Aceria tosichella*	Leaf damage and mite population	–	Richardson et al., 2014
Common bean	*Tetranychus urticae*	Leaf damage, mite population and oviposition rate	–	Tahmasebi et al., 2014
Citrus	*Tetranychus urticae*	Mite oviposition rate	–	Agut et al., 2015
Tomato	*Tetranychus urticae*	Traveled distance on the leaf surface	–	Baier et al., 2015
Tomato	*Tetranychus urticae*	Mite population and oviposition rate	–	Pappas et al., 2015
Apple	*Tetranychus urticae*	Mite population and yield loss	X	Warabieda, 2015
Citrus	*Tetranychus urticae*	Mite oviposition rate	–	Agut et al., 2016
Cherry tomato	*Tetranychus urticae*	Traveled distance on the leaf surface	–	Lucini et al., 2016
Wheat	*Aceria toschiella*	Leaf damage and mite population	–	Aguirre-Rojas et al., 2017
Wheat	*Aceria toschiella*	Leaf damage and mite population	–	Chuang et al., 2017
Rice	*Schizotetranychus oryzae*	Mite survival, development and oviposition rates	–	Gonçalves et al., 2017
Cassava	*Tetranychus urticae*	Leaf damage	–	Liang et al., 2017
Cassava	*Tetranychus cinnabarinus*	Mite survival, development and oviposition rates	–	Lu et al., 2017
Mini tomato	*Tetranychus urticae*	Traveled distance on the leaf surface	–	Maciel et al., 2017
Common bean	*Tetranychus kanzawai*	Leaf damage and mite oviposition rate	–	Ozawa et al., 2017
Maize	*Tetranychus urticae*	Mite survival and development rates	–	Paulo et al., 2017

## Why resistance analysis can be problematic?

Even though the importance of data on plant reproductive success and yield in plant defense studies has been previously emphasized (Clavijo McCormick et al., 2012; Poelman, 2015), the plant-herbivore community still have difficulties to use the appropriate fitness analysis in combination with recent methodological advances to increase our understanding of plant defense traits (Erb, 2018). Therefore, herbivore performance traits and leaf damage—proximate variables, according to Erb (2018)—still are the first options. However, proximate variables has several strong limitations. For example, herbivore population can be a poor predictor of feeding damage (Lu et al., 2015), while herbivore weight gain can be inversely related to herbivore survival (Veyrat et al., 2016). Host plant preference have been widely used to assess plant resistance against herbivores, through the analysis of feeding/oviposition reduction (antixenosis - Stenberg and Muola, 2017). Unfortunately, this is a reliable analysis only for large herbivores, which can directly determine the formation of reproductive structures and plant survival (Huber et al., 2016; Machado et al., 2016; Erb, 2018). For small herbivores such as arthropods, antixenosis as a proximate variable for the study of plant defenses can have limitations, mostly related to the different preferences of adults and juveniles (Clark et al., 2011), difficult to mimic the field situation in lab conditions (Schuman et al., 2015) or the complexity of interaction between the plant and several other herbivores (Kessler and Baldwin, 2004). Leaf damage assessments are often used because they accurately mirror the severity of attack (Erb, 2018). Even though in some cases there is a clear relation between plant damage and yield penalty (Vichitbandha and Chandrapatya, 2011), the relationship between the two variables is not always linear, and many plants can sustain herbivore damage without suffering significant yield penalties through tolerance (Scholes and Paige, 2014; Lehndal and Ågren, 2015; Erb, 2018). We have seen such behaviour in our lab with a rice cultivar infested by *Schizotetranychus oryzae*, in which mite population and leaf damage increased consistently throughout the vegetative and reproductive stages, resulting in no grain yield reduction, while other cultivars showed reductions of more than 60% (unpublished data). Furthermore, recording the exact extent of plant damage caused by most of the herbivores remains challenging and somewhat subjective, due to the need of a visual damage scale, unless quantified in standardized units of leaf area consumed relative to herbivore size (Fragoso et al., 2014) or using image softwares.

## It's time to look at plant tolerance

Another point that favors the more widely exploitation of tolerance mechanisms in crop protection strategies is the fact that antibiosis and antixenosis typically deter herbivore feeding, likely imposing a strong selection pressure on the herbivore to overcome plant resistance (similar to what happen with pesticides). On the other hand, plant tolerance have no effect on herbivore biology or behavior, and therefore is unlikely to impose selection on the herbivore (Mitchell et al., 2016). Thus, plant tolerance is considered a more stable management strategy for pests (Weis and Franks, 2006; Peterson et al., 2017), with greater chance of providing long-lasting pest control (Mitchell et al., 2016). Therefore, based on all these points, we suggest the analysis of plant tolerance mechanisms and yield/productivity in every crop-mite interaction (which could probably be extended to most of the herbivorous pests). In fact, this is what really matters to crop farmers, and academic studies should be aligned with these needs.

It is important to highlight that we are not suggesting the replacement of crop resistance studies by crop tolerance ones. We believe that both defense strategies should be extensively analyzed. We agree with the statement of Peterson et al. (2017): “Before substantial work on tolerance development can occur, we must conduct basic research on the physiological and biochemical mechanisms of tolerance. This must involve interdisciplinary research between plant scientists and entomologists.” On the other hand, in a couple of years we would like to say: no, plant tolerance is not “the forgotten child of plant resistance.” We believe that basic understanding of how plants cope with herbivory and the identification of tolerant genotypes from important crops will have a major impact in pest control and grain yield.

## Author contributions

All authors listed have made a substantial, direct and intellectual contribution to the work, and approved it for publication.

### Conflict of interest statement

The authors declare that the research was conducted in the absence of any commercial or financial relationships that could be construed as a potential conflict of interest.
